# From Eshu to Obatala: animals used in sacrificial rituals at Candomblé "terreiros" in Brazil

**DOI:** 10.1186/1746-4269-5-23

**Published:** 2009-08-26

**Authors:** Nivaldo A Léo Neto, Sharon E Brooks, Rômulo RN Alves

**Affiliations:** 1 Programa de Pós-Graduação em Ciências Sociais, Laboratório de Estudos em Movimentos Étnicos, Universidade Federal de Campina Grande, Avenida Aprígio Veloso, 882-Bloco BA, Bodocongó, 58109-970 – Campina Grande, PB – Brasil; 2 Department of Geography, University of Cambridge, Downing Place, Cambridge CB2 3EN, UK; 3 Departamento de Biologia, Universidade Estadual da Paraíba, Av. das Baraúnas, 351/Campus Universitário, Bodocongó, 58109-753, Campina Grande-PB, Brasil

## Abstract

**Background:**

The practice of sacrifice has occurred in several cultures and religions throughout history and still exists today. Candomblé, a syncretical Afro-Brazilian religion, practices the sacrificial ritual called "*Orô*" by its adherents. The present work aims to document the use of animal species in these sacrificial practices in the cities of Caruaru (PE) and Campina Grande (PB) in Norteastern Brazil, and to further understand the symbolism of these rituals.

**Methods:**

Semi-structured and unstructured interviews and informal discussions were held with 11 Candomblé priests and priestesses between the months of August 2007 and June 2008. We attended rituals performed at "terreiros" where animals were sacrificed, in order to obtain photographic material and observe the procedures and techniques adopted.

**Results:**

A total of 29 animal species were used during sacrificial rituals according to the priests and priestesses. These species were classified in 5 taxanomic groups: Molluscs (n = 1), Amphibians (n = 2), Reptiles (n = 2), Birds (n = 10) and Mammals (n = 14). According to Candomblé beliefs, animals are sacrificed and offered to their deities, known as orishas, for the prosperity of all life. There is a relationship between the colour, sex and behaviour of the animal to be sacrificed, and the orisha to whom the animal is going to be offered. The many myths that form the cosmogony of Candomblé can often explain the symbolism of the rituals observed and the animal species sacrificed. These myths are conveyed to adherants by the priests and priestesses during the ceremonies, and are essential to the continuation of this religion.

**Conclusion:**

Candomblé is a sacrificial religion that uses animals for its liturgical purposes. The principal reason for sacrifice is to please supernatural deities known as orishas in order to keep life in harmony. This is accomplished through feeding them in a spiritual sense through sacrifice, maintaining a perfect link between men and the gods, and a connection between the material world (called *Aiyê*) and the supernatural world (called *Orun*).

## Background

The practice of sacrifice is present in several cultures, and is fundamental to many religions including Jewish, Christian, Islamic and Hindu. Across cultures and civilizations the function of sacrifice is similar; to gain or maintain a connection with a deity through relinquishing something of worth in order to obtain protection or implore a favour [1]. Human sacrifice, for example, was common in some ancient civilizations such as the Egyptian, Chinese, Indian, Aztec, Macedonina, Albanian and some cultures of Ancient Peru [2]. The Aztec indians believed people had a mission to prevent the destruction of the earth through offering human hearts and blood to the god of sun, ensuring the sun would rise the next day [2]. The most famous of examples, however, is perhaps the sacrifice of Christ within the Christian religion for the forgiveness of mankind's sins.

The use of animals in sacrificial practices is common in many cultures and religions, symbolizing the cultural and liturgical value that certain animal species have held throughout history. For instance, in the five books of Moses (Genesis, Exodus, Leviticus, Numbers and Deuteronomy) and in the Old Testament of the Bible, we can find accounts of animal sacrifices, called holocausts, which symbolized offerings to the Christian God for various purposes including gratefulness and forgiveness for unacceptable behavioural deviation or sins.

Sacrificial offerings are regarded as gifts to the gods. Some authors have commented on the transformation process whereby these offerings have to be transformed to enter the arena of the sacred and be received by supernatural deities [3]. Such beliefs have given rise to several rituals surrounding the act of sacrifice. These practices thereby represent a communion between mortals and the divine, and they are believed to result in well-being of some form for all those involved in the offering the animal [4,5]. It has even been suggested by some that the rites of sacrifice are the basis for the survival of society, as a result of bringing communities together, reinforcing interconnectedness, and restoring the harmony disrupted by violence [6,7]. While modern society often condemns sacrificial rituals, they are maintained in many cultures around the world where they are still regarded as important mediators between natural and supernatural worlds [8].

Candomblé is an Afro-Brazilian religion formed by the syncretism of Catholic, African and to some extent Indian faiths, and it is one that involves the practice of animal sacrifice. During the European colonisation of Brazil, enslaved Africans were brought from several countries and tribes of Africa, where great variation in liturgical practices exists and a number of different deities are worshipped. On their arrival in Brazil, there was an "agglutination" process of these religions that, together with the Catholic and indigenous Indian influence, generated the Candomblé Nations, such as the Nagô, the Keto, the Banto, among others. These represent different forms of Candomblé that have originated from different tribes and regions of Africa, and each therefore exhibits a unique set or practices and myths that relates to their African origin. Of importance within this religion are the festivals for the Gods known as Orishas, which principally involves ceremonies of spirit possession by worshippers, animal sacrifices and offerings [9-18]. While there have been some accounts of the sacrificial rituals performed as part of Candomblé worship, there is a paucity of information regarding the type of animals used, and how they relate to the traditional myths that are at the heart of this religion. The present work aims to analyze the use of animal species in Candomblé sacrificial practices in the cities of Caruaru and Campina Grande, in order to understand the symbolism of particular species used in these rituals, thus furthering our understanding of the connection between animals and people in a religious context.

## Methods

### Study area

This study was carried out in two cities situated in the Northeast of Brazil; Caruaru, in the state of Pernambuco, and Campina Grande, in the state of Paraiba. The municipality of Caruaru is in the agreste region of Pernambuco State (8°14'19" S and 35°55'17" W), and is located 136 km from the state capital of Recife. In 2007, the urban population of Caruaru was 217,407, making it the most populous municipality in the Ipojuca Valley, and the total population was estimated to be 289,086 inhabitants [19]. Caruaru is the economic center of the Agreste region of Pernambuco, and of economic significance for the entire Northeast of Brazil. As people from many different areas travel to Caruaru in order to trade, it exhibits a diverse mix of cultures [20].

The city of Campina Grande (7°13'11"S; 35°52'31"W) is located in the Mesoregion of the Agreste Paraibano in Paraiba state, 70 miles away from the state capital city, João Pessoa. The population of Campina Grande in 2007 was estimated to be 371,060 inhabitants [19]. Campina Grande is the second largest municipality in the State of Paraíba, exerting great political and economical influence over 57 other municipalities in the state of Paraíba.

### Data Collection

Field data were collected through semi-structured and unstructured interviews and informal discussions [21-24] with 11 priests and priestesses who originated from the Candomblé nations Keto (n = 5), Iroubá (n = 1) and Nagô/Umbanda (n = 5). These priests and priestesses are popularly known as "pais-de-santo" (fathers of saints) and "mães-de-santo" (mothers of saints), respectively, or Babalorixás and Ialorixás (in the liturgical language used by the adherents of this religion, the Yorubá). They conduct ceremonies in the worship of orishas at a location known as a "terreiro". All those attending who have been initiated into the religion are known as filhos-de-santo (the holy children). Each adherent has a primary orisha who he or she worships, but will participate in the worship of all orishas. Collectively, followers of the Candomblé religion are known as povo-de-santo (the holy people). We gained access to the terreiros through holy sons who the first author previously knew. This facilitated trust, enabling the first author to conduct interviews with priests and priestesses in the first instance. Additional interviewees were chosen by using the snowball technique, based on information initially provided by the first interviewees. Before each interview, the first author introduced himself, explained the nature and objectives of the research, ensured confidentiality of informants and asked the respondents for permission to record the information. Some attempts to interview Candomblé priests and priestesses were unsuccessful due to inaccurate information given regarding their location, and some failed to provide much information because interviewees were reluctant to answer questions. Candomblé rituals are often carried out in secrecy as a result of the antagonism that followers of this religion suffer from a predominantly Christian society, therefore making it challenging to gain access and information regarding these practices. This study was carried out between the months of August 2007 and June 2008. The ethical approval for the study was obtained from the Ethics committee of Universidade Estadual da Paraíba.

The questionnaires were used to gather information on the animal species used in religious ceremonies, forms of use and parts used. The names of the animals were recorded as mentioned by the interviewees, and data was compared with the existing literature on the subject. Consultations with specialists in the groups of animals mentioned by the interviewees were also conducted. Upon authorization, the interviews were taped to provide further confirmation of the information given. Rituals performed at "terreiros" (the physical space where the religious rituals are performed) where animals were sacrificed were attended, in order to observe the procedures and techniques adopted during these rituals as well as to obtain photographic material.

## Results and Discussion

### The symbolic meaning and rites of animal sacrifice

When asked why sacrificial animals were used as offerings to the deities, priests and pristesses were unanimous in their response. They all stated that through the sacrifices the spirits were fortified and fed, and as a result the followers' requests and desires could be met, healing diseases and solving financial and personal problems. According to the interviewees, the energy driven in the form of sacrifice would return as gifts to the practitioners of sacrifice. The following parts of testimonies show the importance of sacrifice and offerings: "*The purpose is one of vitality, of the energy of life. So, when we're offering, when we're sacrificing, we're vitalizing, energizing the contact between man and orisha *(Mother C. of Oshun, 43); "*We give Life for Life. We exchange Life for Life. We're giving treats to the orisha to get positive energy in return *(Father M. of Shango, 46). Sacrifice, is seen as the only way to preserve the harmony that exists among the many components of the natural and supernatural systems [25].

The interviewees employ the expression "to eat" when meaning that a certain spiritual entity will feed itself from the sacrificial offering. They say, for example, that Obatala will "eat" a white nanny-goat. The expression "eat" is used as a symbolism for a spiritual form of feeding. Orishas do not "come down" from the spiritual plain to eat (literally speaking) the animal being offered, but feed off the energy of the offering, energy that Candomblé adherents call by the Nagô word '*Axé'*. *Axé *is characterized as a mystic force that is present in some places, objects or certain parts of the animal body, such as the heart, liver, lungs, genitals, riverbeds, stones, seeds and sacred fruits [12,13,25]. Blood is a vital component in Candomblé as it considered the transporter of the *axé *present in animals [26]. It is therefore always collected and used separately to renew the *axé *of ritual objects [27].

**Figure 1:**
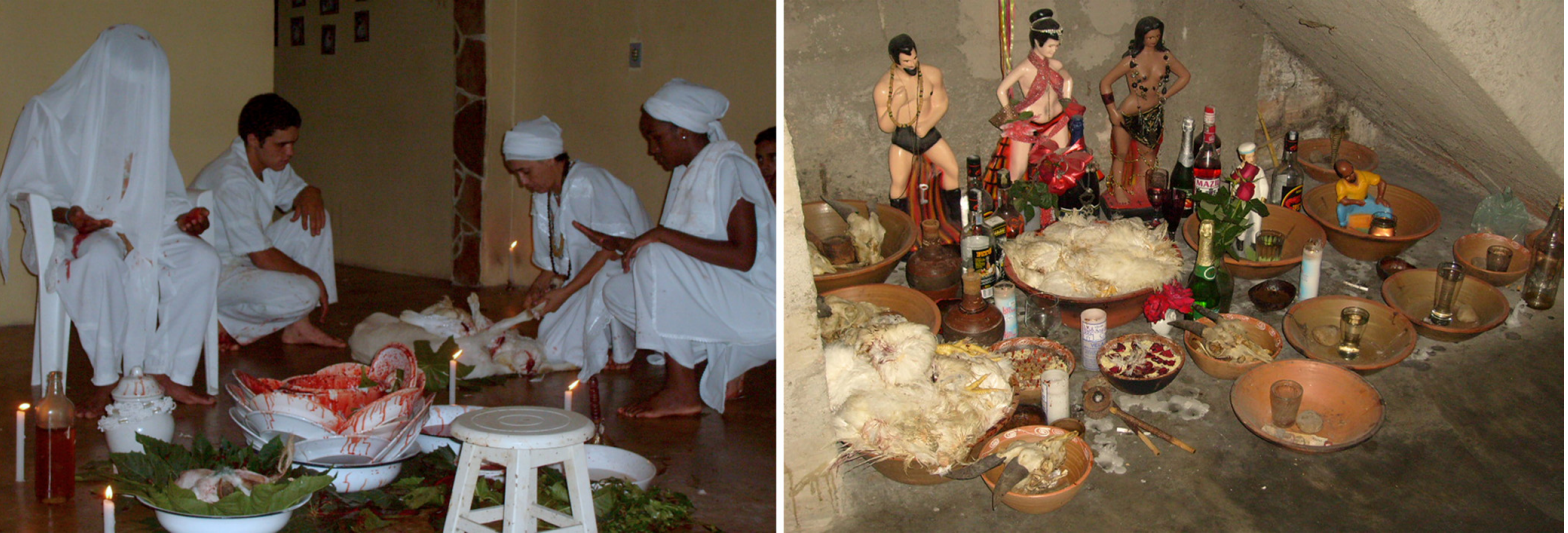
**From the left: Participants of the ritual cut the paws of the nanny-goat to offer them, while the honored orisha (Obatala) has the possession of his "son", sitting on the chair**. At right: Eshu's "assentamento" with offered chickens, goat's brains and drinks.

During Candomble rituals it was observed that, following the sacrifice of an animal, the vital parts thought to be "imbued with *Axé*" are offered to orishas. These parts included the head, paws, wings, liver, gizzard, heart, lungs, liver, genitals, flippers, tail and first ribs. They are first put together and cooked in dende oil, honey and other spices (Figure 1). Dende oil is made from the African oil palm, *Elaeis guineensis*, which is a sacred species to people of African descent. Dende was brought for commercial purposes by Portuguese colonists and were incorporated by Afro-Brazilian healers [26]. The orisha Obatala is an exception in that his offers are cooked only with honey due to his dislike of dende oil. Equally, offerings to Oxossi are not to be mixed with honey. Such aversions shown by orishas are termed 'as quizilas', or 'euó' in yorubá. They usually refer to food, drink and colour and they are regarded as points of weakness for the holy sons (filhos-de-santo) who are forbidden from consuming or wearing them. For example, the holy sons of Iansã are restricted from eating any part of the sheep, and those of Nanã are restricted from wearing the colour purple. These prohibitions do however vary between terreiros, and the nature of them is often held secret as it is believed that rival priests can threaten the axé of other terreiros [26].

According to the interviewees, the animal parts, once prepared, are placed in a wooden or ceramic container and thereafter wrapped with tissue from the nanny-goat's (*Capra hircus*) stomach (called *Axó*, lieterally meaning "*Axé*'s clothes). *Axó *prevents negative energy from other undesired entities entering the offering and draining its vitality. The offering is subsequently placed at the "saint's feet", which means in the place devoted to each orisha, called '*assentamento*' or '*Ibá' *(Figure 1).

A few days after the sacrificial ritual, a communal feast takes place whereby the other parts of the animal that are not offered are used to prepare several dishes and given to the people present at the festival. As pointed out by Nadalini [28], the Candomblé "communal feast" signifies a link between men and the deities. According to Santos [25], the *Axé *is a transmissible force whereby it can be passed between all the material and supernatural presence at the "*terreiro*". The consumption of the meat of an offerred animal that has *Axé*, is a way of starting a communion with the gods [4,5], whereby the followers would be sharing the "same" food their own gods enjoy. However, the meat of some animals cannot be consumed. According to Mother C. of Oshun, Ialorixá of the Keto Nation, some animals, like the pig (*Sus scrofa*), have *quizila*, a negative energy, and the follower is forbidden to eat that meat, which is then donated to communities in need.

Communal feasting plays a prominent part in Candomblé as it does in many other religions, including Christianity whereby Holy Communion signifies eating the body and drinking the blood of Jesus Christ. In addition to the strong associations formed between the deities and their followers through these sacrificial practices, communal feasting reinforces the interconnectedness of the community of adherants, and therefore plays a significant role in the maintenance of the Candomblé religion.

### The liturgical requirements of animals used in sacrificial rituals

A total of 29 animal species were mentioned by the priests and priestesses. The species were classified in 5 taxonomic groups: Molluscs (n = 1), Amphibians (n = 2), Reptiles (n = 2), Birds (n = 10) and Mammals (n = 14). While most of the species listed are not considered to be of conservation concern, the yellow-footed tortoise (*Chelonoidis denticulata*) is listed as Vulnerable to extinction by the World Conservation Union (IUCN) (Table 1). All of these species are used in sacrificial rituals called *Orô *by followers of the Candomblé. The type of animal used depends on the orisha to whom the offering is made. While some species may be sacrificed in honour of more than one orisha, others are the species of preference for particular orishas, and considered 'major treats'. Some species serve particular functions, such as 'cleaning' whereby they are oferred to orishas as part of a healing ritual, or are used to perform Bori, a type of initiation ritual.

**Table 1:** Animals used as sacrificial offerings at Candomblé terreiros in the visited cities.

**Family/Species**	**Local and English Name**	**IUCN category**	**Deity offering made to**	**Number of mentions**	
				
				CG	CA
**MOLLUSCS**					

Achatinidae					
*Achatina fulica *(Ferussac, 1821)	*Ibi*, *Igbin *or Boi-de-Oxalá (snail)		Obatala	---	6

**AMPHIBIANS**					

Family not identified					
*Species not identified*	Rã-de-peito (frog)		Major "treat" to Oshun	---	1
Bufonidae					
*Rhinella schneideri *(Werner, 1894)	Sapo-boi (toad)	LC	Major "treat" to Nana	---	1

**REPTILES**					

Chelidae					
*Phrynops geoffroanus *(Schweigger, 1812) – "cágado"	Cágado d'água (Geoffroy's side-necked turtle)		Shango	---	2

Testudinidae					
*Chelonoidis denticulata *(Linnaeus, 1766)	Jabuti (yellow-footed tortoise)	VU	Shango	2	7

**BIRDS**					

Anatidae					
*Anas *sp.	Pata (duck)		Oshun and Yemaja	---	4
Columbidae					
*Columba lívia *(Gmelin, 1789)	Pombo, *Irilé *(pigeon)		To perform Bori. Orishas; "Cleaning"	2	8
*Columbina sp.*	Rolinha (ground dove)		Oxossi	2	2
*Zenaida auriculata *(Des Murs, 1847)	Ribaçã (eared dove)	LC	Oxossi	2	2

Meleagrididae					
*Meleagris gallopavo *Linnaeus, 1758	Peru (turkey)		Iansan; Logun-edé; Oxossi	1	5

Numididae					
*Numida meleagris *(Linnaeus, 1758)	Guiné, Galinha-d'angola, *Coquém *(helmeted guineafowls)	LC	To perform Bori	2	7

Phasianidae					
*Gallus gallus *(Linnaeus, 1758)	Galinha (chicken)		Orishas; "Cleaning"	2	7
*Pavo cristatus *Linnaeus, 1758	Pavão (peacock)		Logun-Edé; Oxossi	1	5
*Phasianus sp.*	Faisão (pheasant)		Logun-Edé; Oxossi	---	5

Tinamidae					
*Nothura boraquira *(Spix, 1825) LC	Codorna (tinamou)		Oxossi	1	2

**MAMMALS**					

Agoutidae					
*Agouti paca *(Linnaeus, 1766)	Paca	LC	Oxossi	---	1

Bovidae					
*Bos taurus *Linnaeus, 1758	Boi/Vaca (ox/cow)		Orishas; Xangô-Aganjou; Exu de Ganga; to perform Bori	2	5
*Bubalus bubalis *(Linnaeus, 1758)	Búfalo (buffalo)		Orishas; except for Iansan	---	4
*Capra hircus *Linnaeus, 1758	Bode/Cabra (goat/nanny-goat)		Orishas	2	7
*Ovis aries *(Linnaeus, 1758)	Carneiro/Ovelha (sheep)		Shango	1	7

Canidae					
*Canis lupus *(Linnaeus, 1758)	Cachorro (dog)		A manifestation of Ogun (Ogunjá) in Africa	1	5

Caviidae					
*Cavia aperea *Erxleben, 1777	Preá (guinea pig)	LC	Oxossi	2	2

Cervidae					
*Mazama americana *(Erxleben, 1777)	Veado-do-mato (red brocket)	DD	Oxossi; also worshiped alive as a sacred animal	---	5

Dasypodidae					
*Dasypus novemcinctus *(Linnaeus, 1758)	Tatu (Nine-banded armadillo)	LC	Obaluaê/Omolu	1	3
*Euphractus sexcinctus *(Linnaeus, 1758)	Peba (Six-banded armadillo)	LC	Shango; Obaluaê/Omolu	2	2

Erethizontidae					
*Coendou prehensilis *(Linnaeus, 1758)	Porco-espinho (porcupine)	LC	Oxossi	---	1

Leporidae					
*Oryctolagus cuniculus *(Linnaeus, 1758)	Coelho (rabbit)	NT	Oxossi	1	3

Suidae					
*Sus scrofa *(Linnaeus, 1758)	Porco (pig)		Oxossi; Omolu. To perform Bori.	2	5

Tayassuidae					
*Pecari tajacu *Linnaeus 1758	Porco-do-mato (peccary)	LC	Oxossi	---	1

Legends: CA (Caruaru – Pernambuco); CG (Campina Grande – Paraíba)

Note: Categories of IUCN- LC (Least Concern), DD (Data Deficient), NT (Near Threatened), VU (Vulnerable).

According to the informants, domestic animals like nanny-goats (*Capra hircus*), chickens (*Gallus gallus*), helmeted guineafowls (*Numida meleagris*) and pigeons (*Columba livia*) are used most often. While wild animals like the yellow-footed tortoise (*Chelonoidis denticulata*) and the red brocket (*Mazama americana*) are used, it is far more restricted. Two factors contribute to the priest's preference for domestic animals: 1) the difficulty in acquiring wild species due to prohibition by environmental law and 2) the sacred symbolism of some species, which implies its protection by the Candomblé adherents.

Each orisha has a specific abode, a kingdom which he or she governs and in which he or she resides. Oxossi, for example, is considered to be a hunter who reigns over the wild forests. As this orisha is regarded as a protector of wildlife, wild animals such as the red brocket (*Mazama americana*) are not used in sacrificial rituals in his honour. When a certain deity requests an animal that is difficult to find, the priest or priestess establishes a communication channel with the deity that made the request. This consultation consists of explanations for failing to provide certain offerings and negotiations regarding alternatives. It is carried out using four cowrie-shells that are thrown to the ground by the priest or priestess, who then interprets the will of the deities by the way the shells fall.

Animals that are used as sacrificial offerings are required to be healthy, fair, strong and without any physical problems. The sex of the animal is also relavent and related to the gender of the orishas. Female orishas (*Iabás*) "*eat*" female animals, while male Orishas (*Borós*) "*eat*" male animals. There is an exception to this rule. The orisha Obatala is the only male orisha who "*eats*" in the *Iabás *circle, thus accepting sacrifices of female animals in his honor. Bastide [9] commented on the androgynous characteristics of Obatala as an explanation of why this orisha accepts female animals as offerings. According to some priests, however, Obatala does not have a sex, since, according to the myths, he is the Father of Creation. Obatala is therefore the equivalent of God in the catholic syncretism who also does not have a specific sex.

**Figure 2:**
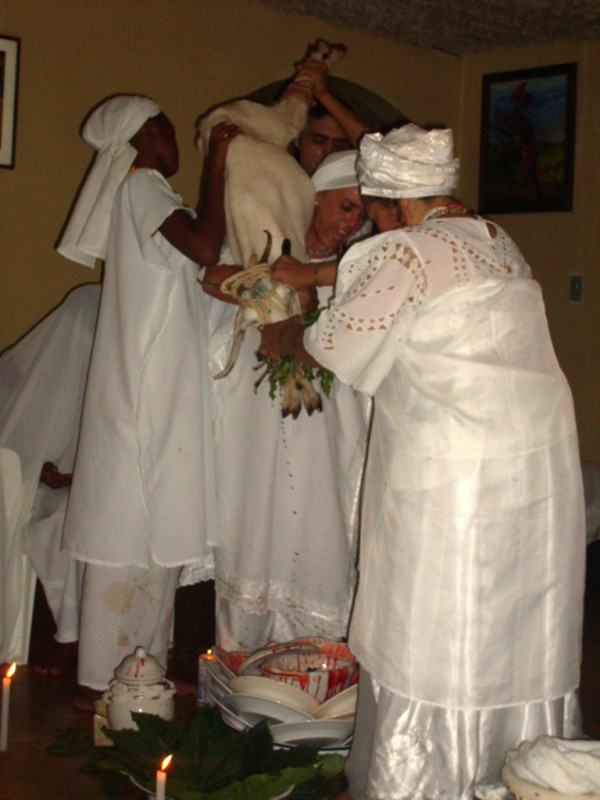
**White nanny-goat being sacrificed in Obatala's honor**.

**Figure 3:**
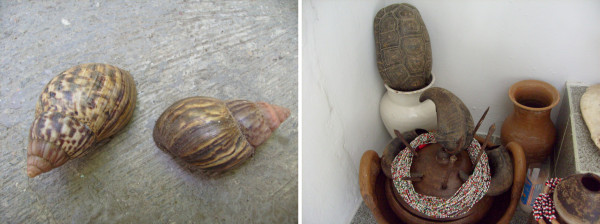
**On the left: Giant African land snail (*Achatina fulica*), known as *Igbin *or boi-de-Oxalá; On the right: shell of the yellow-footed tortoise (*Chelonoidis denticulata*) and the horns of the buffalo (*Bubalus bubalis*) being offered in sacrifice**.

**Figure 4:**
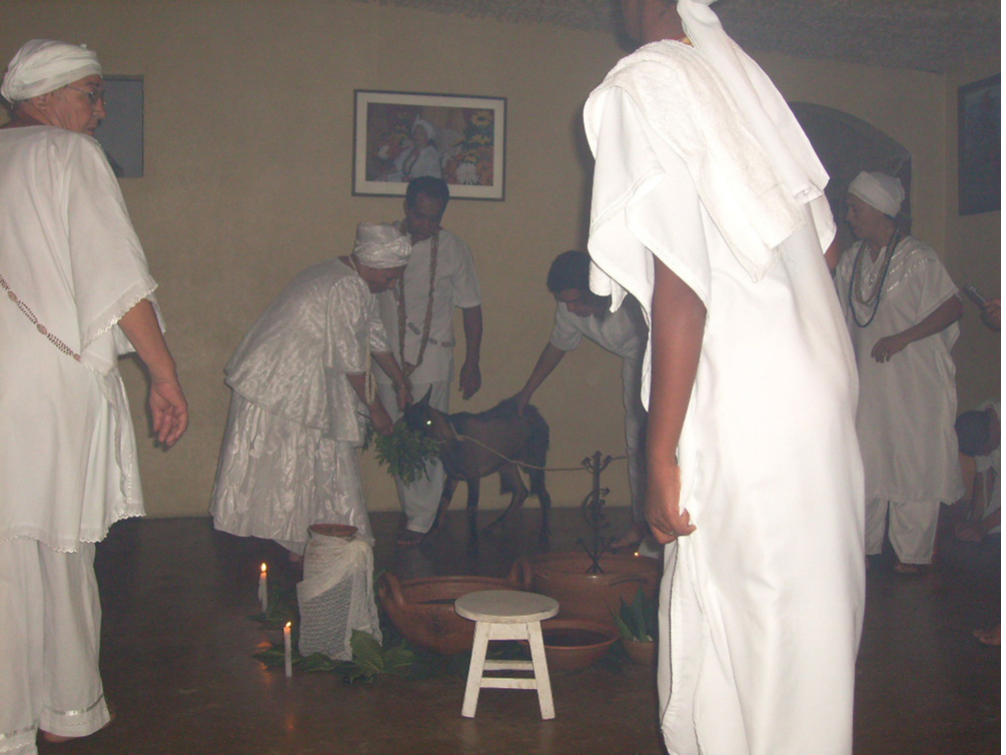
**A dark goat being presented to everybody present at the ritual and, especially to orishas**. This animal was sacrificed in Eshu's honor.

Color is also an important criterion for offerings to the orishas. Each orisha has a color that symbolizes him or her, and this color is present in the necklace, known as *guias*, worn by the holy people ("povo-de-santo"). The color may vary between different Candomblé Nations and represents characteristics of an orisha's personality and the elements that symbolises them. For example red signifies fire and fury, white – tranquility and age, and yellow – prosperity and wealth. The color preference of the orisha determines the color of the animal that will be sacrificed in his or her honour. For example, for sacrificial offerings to Obatala, considered an *orixá-funfun *(literally "white orisha"), the animals or their parts should be completely white (Figure 2), such as the white blood of the mollusk called *Igbin *(*Achatina fulica*) (Figure 3). Those offered to Eshu's should preferencially be dark in colour, such as a black goat (*Capra hircus*) (Figure 4).

Behavioral characteristics of the animal to be sacrificed are also often related to the personality of the orisha to whom they are offerred. Examples are the graceful swimming style of the duck (*Anas *sp.) that is offered to Oshun, who is considered to be vain, fair and seductive, and the strength and resistance of the tortoise (*Chelonoidis denticulata*) that is offered to Shango who is considered to be strong and powerful (Figure 3). The offered items thereby carry the symbology of the supernatural deity to whom they are offered, allowing for the restoration of the energy, elements and function represented by each Orisha [25].

Some animal uses for religious purposes in Brazil are similar to those in Africa, indicating that these rituals are likely to have originated in Africa. In the dry regions of Nigeria, animal products are used in cultural ceremonies (e.g. for funerals or when leaders take office), in traditional rites (e.g., to invoke or reconcile with the Gods), and have a very significant role in the traditional pharmacopoeia [29]. Some of the animal species observed in sacrificial rituals in this study are also present in Africa, and there was a sense among priests that species present in Africa have a special level of axe, or vital energy. While some species used in Brazilian Candomblé, such as the Helmeted Guineafowl, *Numida Meleagris*, are also used in sacrificial rituals in African countries, other species are substitutes for African species that do not occur in Brazil. For example *Achatina fulica *is used in Brazil as a substitute for the African Giant Snail (*Archachatina marginata*) that is used in Nigeria. Although they are different species, they are known by the same name (igbin) in both Brazil and Nigeria [30].

### Sacrificial procedures and techniques and the role of myths

In Candomblé, the physical position of actors within the 'terreiro' as well as their function within the ritual is of importance and is highly respected by followers of this religion. For example, the person in charge of the sacrifice of animals, known as *Ashogun*, always stands in a specific position within the terreiro. According to the interviewees, the *Ashogun *has to be a man and he must be initiated to the worship of Ogun, the patron orisha of steel, iron and ores and the owner of the *obé *of steel (knife). Only men can perform the function of sacrifice, since women are the givers, not the takers of life, as stated by the following priests:

Woman gives life. The woman is born to give life. As the Obatala story, Father of Creation, the woman generates, the woman gives the Life, so she cannot take it in our Religion (Father M. of Shango, 46).

The woman was born to create, not to destroy (Father J. of Ogun, 46).

The killing methods depend on both the type of animal used and the orisha to whom the animal is offerred. Animals considered to be sacred, such as the *Coquém *and the *Irilé*, yorubá names attributed respectively to the helmeted guineafowls (*Numida meleagris*) and the pigeon (*Columba livia*), are not killed by the knife. In this case, leaves of Saião (*Kalanchoe sp.*), are used to strangle and decapitate the bird. The helmented guineafowl is believed to be the first terrestrial animal created and is therefore symbolic as a representation of the creation of the world. The pigeon is believed to be a messenger for the gods, and thereby a way of informing the gods of the ceremony taking place. These animals are also used in the initiation ceremonies known as *Bori *that takes place when an individual accepts Candomblé as their religion and agrees to follow the associated traditions, such as participating in sacrificial rituals and conforming to the preferences of their chosen orisha.

The myths of Candomblé, known as '*Odu*' are central to the religion as a way of maintaining the traditions and practices involved in worship through oral transmission. For this reason they can explain the procedures and techniques adopted during sacrificial rituals [10]. One of the primary functions of a priest is to share the wisdom of '*Odu*' with adherents that should therefore, not be interpreted scientifically, but seen as a way to revive the primordial mentality and maintain religious practices [31]. Almost all of the myths within Candomble originate from African oral traditions, once again highlighting the strong connection between this religion and its African forebearers.

Orishas are considered to be temperamental and capable of human feelings such as envy, jealousy, anger and love. For this reason, myths about fights between orishas for a variety of different reasons are common, and often explain the differences observed among rituals for different orishas. For example, while offerings to almost all orishas are performed using the *obé *of steel, those made to Nanã and Omolu are not, as explained by the following myths:

Ogun challenged Nanã, saying that nobody would receive the worship, because no one could receive the worship without receiving Ogun's energy. That is why no orisha should be worshipped without worshiping Ogun, because Ogun is the owner of the knife, the owner of the obé, the owner of the iron. And Nanã challenged him. And said yes, there would be sacrifice to her, and yes, there would be worship to Nanã without using the obé (Mother C. of Oshun, 43).

Nanã ensured him that she was capable of surviving without him. So the worship to Nanã cannot have anything ruled by Ogun, for example, iron, steel, ore, etc. (Father M. of Shango, 46).

**Figure 5:**
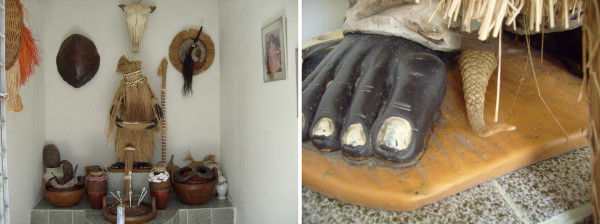
**"Assentamento" or *Ibá *(saint's feet) of the orisha Omolu where the offering is placed**. On the right, the tail of a nine-banded armadillo (*Dasypus novemcinctus*) being offered in sacrifice.

In order to worship Nanã the sacrifice of animals is performed using either a sharpened stone implement, an *obé *made of wood, a capim-navalha (a type of sedge grass that cuts like a razor) or even glass. Glass is considered appropriate as it is formed from sand, and Nanã herself is associated with earth and clay. The reason why the worship to Omolu does not use the steel *obé *is that, according to the myths, Omolu is the son of Nanã and, out of respect to his mother, nothing from Ogun's domain, including the steel *obé*, is used in his worship (Figure 5).

### Healing through sacrifice

Within the scope of sacrificial practices, there are certain rituals that are used to heal diseases [32-42]. Bastide [9] uses the term "heads exchange" for this type of ritual as it is often believed that the disease of the human being is exchanged for the animal's health, usually a rooster or a chicken (*Gallus gallus*). The live animal is passed over the sick person's body, allowing the human disease to pass to the animal. The animal is subsequently killed exterminating the evil that was inflicting the human being. Some priests in this study, however, believe differently. They do not believe that the disease is passed to the animal, but that the sacrifice is an offering to the orisha known as Omulu who is responsible for healings and who, in return, would comply with the healing requests made during the ritual.

## Conclusion

Candomblé is an Afro-Brazilianreligion that is based on African traditions and practiced chiefly in Brazil. Rituals based on animal sacrifice form a central part of the ceremonies held in worship of the gods known as Orishas. The types of animals used and their mode of sacrifice are supported by beliefs and myths associated with the religion, and depend upon the preferences of the orisha to whom they are offered. As the myths and practices associated with Candomblé originated in Africa, some of the animal species used in sacrificial rituals either also occur in parts of Africa, or are substitutes for African species. The principal reason for sacrifice is to please the orishas in order to keep life in harmony. This is accomplished through feeding them in a spiritual sense through sacrifice, maintaining a perfect link between men and the gods and a connection between the material world (called *Aiyê*) and the supernatural world (called *Orun*).

Domestic animals are mostly prefered for sacrificial purposes as wild animals are often considered sacred by adherants of the religion or are protected by environmental laws. Of the wild species used, only the yellow-footed tortoise (*Chelonoidis denticulata*) is considered to be threatened with extinction according to the IUCN red list of endangered species. These practices, compared to many other human uses of wildlife, are therefore not of serious conservation concern. The value of animals to humans for food, income and medicine is often reported, but their importance in a religious context is far less recognised. The incorporation of animals in long-standing cultural and religious practices, such as this, signifies the spiritual value that many animal species have held throughout history until the present day.

## Competing interests

The authors declare that they have no competing interests.

## Authors' contributions

NALN, RRNA, SEB – Writing of the manuscript, literature survey and interpretation; NALN – Ethnozoological data; RRNA – Analysis of taxonomic aspects. All authors read and approved the final manuscript.
